# Selective Inactivation of PTEN in Smooth Muscle Cells Synergizes With Hypoxia to Induce Severe Pulmonary Hypertension

**DOI:** 10.1161/JAHA.113.000188

**Published:** 2013-06-21

**Authors:** Henrick Horita, Seth B. Furgeson, Allison Ostriker, Kyle A. Olszewski, Timothy Sullivan, Leah R. Villegas, Michelle Levine, Jane E. Parr, Carlyne D. Cool, Raphael A. Nemenoff, Mary C. M. Weiser‐Evans

**Affiliations:** 1 Division of Renal Diseases and Hypertension, Department of Medicine, University of Colorado, Anschutz Medical Campus, Aurora, CO (H.H., S.B.F., A.O., K.A.O., M.L., R.A.N., M.M.W.E.); 2 Division of Pulmonary Sciences and Critical Care, Department of Medicine, University of Colorado, Anschutz Medical Campus, Aurora, CO (J.E.P., C.D.C.); 3 Division of Cardiovascular Pulmonary Research Program, Department of Medicine, University of Colorado, Anschutz Medical Campus, Aurora, CO (T.S., L.R.V., R.A.N., M.M.W.E.)

**Keywords:** hypoxia, inflammation, pulmonary hypertension, remodeling, smooth muscle

## Abstract

**Background:**

Pulmonary vascular remodeling in pulmonary hypertension (PH) is characterized by increased vascular smooth muscle cell (SMC) and adventitial fibroblast proliferation, small vessel occlusion, and inflammatory cell accumulation. The underlying molecular mechanisms driving progression remain poorly defined. We have focused on loss of the phosphatase PTEN in SMCs as a major driver of pathological vascular remodeling. Our goal was to define the role of PTEN in human PH and in hypoxia‐induced PH using a mouse model with inducible deletion of *PTEN* in SMCs.

**Methods and Results:**

Staining of human biopsies demonstrated enhanced inactive PTEN selectively in the media from hypertensive patients compared to controls. Mice with induced deletion of *PTEN* in SMCs were exposed to normoxia or hypoxia for up to 4 weeks. Under normoxia, SMC *PTEN* depletion was sufficient to induce features of PH similar to those observed in wild‐type mice exposed to chronic hypoxia. Under hypoxia, *PTEN* depletion promoted an irreversible progression of PH characterized by increased pressure, extensive pulmonary vascular remodeling, formation of complex vascular lesions, and increased macrophage accumulation associated with synergistic increases in proinflammatory cytokines and proliferation of both SMCs and nonSMCs.

**Conclusions:**

Chronic inactivation of *PTEN* selectively in SMC represents a critical mediator of PH progression, leading to cell autonomous events and increased production of factors correlated to proliferation and recruitment of adventitial and inflammatory cells, resulting in irreversible progression of the disease.

## Introduction

The pathogenesis of pulmonary hypertension (PH) is multifactorial, with vasoconstriction and vascular remodeling contributing to increased pulmonary vascular resistance leading to right heart failure.^1–6^ A hallmark of vascular remodeling in PH involves vascular cell proliferation contributing to thickening of elastic and muscular pulmonary arteries and muscularization of small peripheral pulmonary arteries. Pulmonary vascular remodeling is an important component of various types of human PH, including idiopathic and familial forms of PH, as well as experimental animal models, including hypoxia‐induced PH.^5,7^ However, unlike humans, in whom PH progresses to a largely irreversible pathology, chronic hypoxia‐induced PH in all animal models is reversible upon return to normoxic conditions.^7^ Remodeling is characterized by medial and adventitial hypertrophy due to increased vascular smooth muscle cell (SMC) proliferation and adventitial accumulation of fibroblasts and myofibroblasts.^1,7^ In addition, intimal changes associated with endothelial dysfunction, including intimal thickening and vessel occlusion, are often observed in human PH.^3,8^ Functional changes in resident vascular cells promote pathological remodeling through dedifferentiation and induction of a promigratory, proproliferative SMC phenotype and activation of adventitial fibroblasts, which proliferate, differentiate to a myofibroblast phenotype, and produce extracellular matrix proteins. Recent data suggest inflammation also plays a significant role in the pathogenesis of human PH and in experimental animal models of PH.^9–11^ For example, chronic hypoxia results in enhanced lung production of specific cytokines, including monocyte chemotactic protein‐1 (MCP‐1), Interleukin 1β (IL‐1β), Interleukin 6 (IL‐6), and stromal cell‐derived factor 1 α (SDF‐1α), leading to the recruitment and accumulation of proinflammatory cells to the remodeling pulmonary vasculature.^9–10,9–13^ However, the molecular mechanisms and signaling pathways underlying lung vascular cell cytokine production and proinflammatory cell recruitment during the pathogenesis of hypoxia‐induced pulmonary vascular remodeling have yet to be defined.

Major advances have identified numerous factors involved in this highly complex pathobiology, however the underlying event(s) initiating the onset of PH are, as yet, not clearly defined. While germline mutations in the *BMPR2* gene have been identified in a large percentage of patients with familial PH,^14–15^ such mutations are absent in the majority of patients with idiopathic forms of PH, suggesting additional mechanisms play an important role in the pathogenesis of PH. We have focused on the essential role of the tumor suppressor, *PTEN*, specifically in vascular SMCs, in the regulation of pathological vascular remodeling. *PTEN* is a dual‐specificity protein and lipid phosphatase that suppresses multiple signaling networks involved in cell proliferation, survival, and inflammation. Tight regulation of *PTEN* is essential for the maintenance of normal physiological states.^16–18^ *PTEN* classically functions as a cytoplasmic lipid phosphatase that dephosphorylates Phosphatidylinositol (3,4,5)‐trisphosphate (PIP3) thereby antagonizing Phosphatidylinositide 3 (PI3)‐kinase/protein kinase B (Akt)‐mediated signaling events.^19–23^ In addition, *PTEN* dephosphorylates protein substrates, such as focal adhesion kinase (FAK), members of the mitogen‐activated protein kinase (MAPK) signaling pathway, and the PI3‐kinase p85 subunit thus underlying its importance in the regulation of multiple cellular functions.^24–27^ *PTEN* is susceptible to positive and negative transcriptional regulation, posttranscriptional inhibition through specific microRNAs, and posttranslational regulation by reversible phosphorylation and oxidation leading to inactivation of its phosphatase activity.^20,28^ At present, no functional redundancy for PTEN has been identified. Although PTEN's role in cancer progression has been extensively studied, accumulating data support an essential role for *PTEN* in normal physiological functions. Our group and others have demonstrated that regulation of *PTEN* in SMCs is critical in pathological vascular remodeling.^29–34^ Notably, *PTEN* negatively regulates SMC phenotypic modulation with loss resulting in multiple downstream events regulating injury‐induced neointima formation. In the systemic vasculature, we showed that molecular depletion of *PTEN* in SMCs promotes massive intimal lesions similar to human restenotic lesions.^33^ PTEN‐associated changes include cell autonomous events resulting in induction of a proliferative, inflammatory phenotype, as well as nonautonomous events leading to recruitment and activation of inflammatory cells. However, much less is known regarding the role of SMC‐derived PTEN in the pathogenesis of PH. We previously demonstrated that cardiovascular depletion of *PTEN* resulted in pulmonary vascular remodeling consistent with pulmonary hypertension.^34^ In addition, Ravi, et al^35–36^ reported dysregulation of *PTEN* in monocrotaline‐ and hypoxia‐induced PH as well as in PH secondary to left heart failure supporting an important role for *PTEN* in SMCs on PH progression. However, the direct role of SMC *PTEN* inactivation on the progression of PH is unclear.

The emerging critical role for SMCs in initiating and promoting vascular disease suggests that targeting SMCs could be a potent strategy to inhibit pulmonary vascular remodeling and PH progression. We hypothesized that SMCs are central initiators of pulmonary vascular remodeling whereby *PTEN* inactivation promotes resident pulmonary vascular cell proliferation as well as recruitment of inflammatory cells through paracrine mechanisms. In this report, we found a marked increase in expression of phosphorylated, inactive *PTEN* in the pulmonary vasculature of lung tissues from a small cohort of pulmonary hypertensive patients compared to normal lung tissue, providing a rationale to further examine chronic SMC‐selective inactivation of *PTEN* on PH progression. Our previous work demonstrated that constitutive depletion of *PTEN* in SMCs was associated with histopathology consistent with PH.^34^ However, these studies were conducted using *PTEN*^LoxP/LoxP^ mice crossed to mice expressing Cre recombinase under the control of the SM22α promoter. Early perinatal lethality precluded an assessment of PH progression. In addition, since SM22α is transiently expressed in cardiomyocytes in early development and this was a constitutive system, we could not rule out the possibility that pulmonary vascular changes were due to secondary effects of cardiac abnormalities or from developmental defects. Therefore, to explore the direct role of SMC‐specific *PTEN* in the progression of PH, we generated inducible, SMC‐specific *PTEN* mutant mice (*PTEN* iKO), expressing the Rosa26 reporter for fate‐mapping SMCs. We demonstrate that inactivation of SMC *PTEN* synergizes with hypoxia to promote an irreversible progression of PH characterized by increased pressure, extensive pulmonary vascular remodeling with formation of complex vascular lesions, and considerable macrophage accumulation.

## Materials and Methods

### Animals and Generation of *PTEN* Mutant Mice

Seven‐week‐old male Sprague‐Dawley rats (Harlan) were housed in a hypobaric chamber for 3 weeks at a simulated altitude of 17 000 ft. (Pb=410 mm Hg; inspired PO_2_=76 mm Hg). Control rats were kept at Denver ambient pressure (5280 ft.;Pb=630 mm Hg, inspired PO_2_=122 mm Hg). *PTEN*^flox/flox^ mice (Dr. Tak Mak, Ontario Cancer Institute, University of Toronto, Toronto, Ontario, Canada), smooth muscle myosin heavy chain (SMMHC)‐CreER^T2^ transgenic mice (Dr. Stefan Offermanns, University of Heidelberg, Heidelberg, Germany), and ROSA26 reporter (R26R) mice (Jackson Laboratory) were bred to generate tamoxifen‐inducible SMC‐specific PTEN knockout mice carrying the R26R allele (PTEN iKO), as described previously.^33^ Controls expressed SMMHC‐CreER^T2^ and R26R but were wild type (WT) for PTEN. Mice received 1‐mg IP tamoxifen injections for 5 consecutive days starting 8 days before hypoxia exposure. Tamoxifen given prior to the experimental protocol genetically and permanently marked differentiated SMMHC‐expressing SMCs through Cre‐mediated β‐galactosidase (βGal) knock‐in and, in *PTEN* iKO mice, deletion of *PTEN* in these cells. Following Cre induction, mice were housed at Denver ambient pressure or in a hypobaric chamber for up to 28 days. At the indicated times, right ventricular systolic pressures (as a measure of pulmonary arterial pressure) were obtained, as described below. Twenty‐four hours prior to sacrifice, mice received 2 IP injections of BrdU (10 mg/kg body weight) 12 hours apart. A minimum of 6 mice per group were used for all experiments; 8‐week‐old (at the start of tamoxifen injections) male mice were used. Rats and mice were housed in the Center for Comparative Medicine, and procedures were performed under a protocol approved by the Institutional Animal Care and Use Committee at the University of Colorado Denver Anschutz Medical Campus.

### Right Ventricular Systolic Pressure and Right Ventricular Hypertrophy Measurements

Mice were anesthetized by inhaled isofluorane (2% to 4%) mixed with room air (21% oxygen, 79% nitrogen). Right ventricular systolic pressure (RVSP) was measured by direct RV puncture in a closed chest. A 25‐gauge needle attached to a DTX Plus pressure transducer (Becton Dickinson) was introduced into the RV and live pressure tracings were measured using the Cardiomax III Cardiac Output program (Columbus Instruments). Pressures were monitored for at least 30 seconds, and averaged every 10 seconds to account for beat‐to‐beat variability. Right ventricular hypertrophy (RVH) was calculated using the following formula: right ventricle (RV) weight/left ventricle (LV)+septum (S) weight.

### Human Lung Tissue

Decoded and deidentified archival lung tissue sections from patients diagnosed with PH (autopsy or transplant; PH with CREST, PH with congenital heart disease, PH with collagen vascular disease, PH with scleroderma/CREST, PH associated with cirrhosis, primary PH; n=6) or failed organ donor controls (n=3) were obtained from Dr. Cool and were stained for phospho‐PTEN and smooth muscle alpha actin as described below. Use of these tissues for this study was reviewed by the University of Colorado COMIRB and approval obtained as no human subjects; patient clinical data were not available for these tissues.

### Morphometry and Immunohistochemistry

Formalin‐fixed, paraffin‐embedded tissues were analyzed by H&E staining for morphology. Sections were visualized using an Olympus light microscope and measurements made using SPOT software. A minimum of 5 mice per genotype were analyzed for vessel wall thickness and muscularization of microvessels. Average vessel wall thickness was calculated using the following formula: (vessel circumference/2π)−(lumen circumference/2π). For immunohistochemistry, formalin‐fixed, paraffin‐embedded tissues were deparaffinized, rehydrated and underwent antigen retrieval by heating for 20 minutes at 115°C in a decloaking chamber (Biocare). Antigen:antibody complexes were visualized using kits from Vector Laboratories and sections lightly counterstained with hematoxylin. Sections immunohistochemically stained for BrdU incorporation were pretreated with 2N HCl to denature DNA. For double labeling, sections were sequentially incubated with specific primary and secondary antibodies followed by selective substrates (Vector). Antibodies used include monoclonal antibromodeoxyuridine (BrdU;1:100; BD Pharmingen), polyclonal anti‐smooth muscle alpha actin (αSMA; 1:1000; Abcam), polyclonal anti‐Von Willebrand Factor (vWF; 1:1000, Abcam), polycolonal antiphospho PTEN (1:100, Novus), and monoclonal anti‐Mac‐3 (1:50; BD Pharmingen). Negative controls included the use of rat or rabbit IgG. To stain for LacZ activity, tissues were fixed in glutaraldehyde and whole mount staining was performed at 37°C overnight using a kit from GTS, Inc according to the protocols provided. Tissues were then paraffin‐embedded for histological analysis. To quantify in vivo replication rates, αSMA‐positive and αSMA‐negative cells were analyzed independently for BrdU‐positive nuclei; total numbers of BrdU‐positive nuclei were determined by counting 30 to 35 vessels per condition from a minimum of 4 animals per genotype. Macrophage accumulation was quantified by similarly counting Mac3‐positive cells. The number of partially and fully muscularized distal pulmonary vessels was counted in α‐SMA‐positive stained sections; five 10×‐power fields from each stained section were counted.

### Quantitative Real‐Time PCR

To assay for cytokine mRNA expression, total RNA was isolated from whole lung tissues by first digesting in RLT lysis buffer (Qiagen) using gentleMACS tubes (Miltenyibiotec). Samples were then processed with QIAshredder and RNeasy Plus kits (Qiagen) to isolate RNA. First strand cDNA was made using the iScript cDNA synthesis kit (BioRad). Sequence‐specific primers were designed: SDF‐1α: forward (5'‐ CTTGTCTGTTGTTGCTTTTCAGCC‐3'), reverse (5'‐ GCCAGAGC‐CAACGTCAAACATC‐3'); IL‐6: forward (5'‐GGTCCTTAGCCACTCCTTCTGTG‐3'), reverse (5'‐GATGCTACCAAACTGGATATAATC‐3); β‐Actin: forward (5'‐ AGGGTGTGATG GTGGGTATGG‐3'), reverse (5'‐AATGCCGTGTTCAATGGGG‐3'). Quantitative real‐time PCR was performed as previously described^29,33,37^ and β‐actin was used for normalization.

### Immunoblotting

Whole lung tissues were harvested, snap frozen in liquid nitrogen, and digested and lysed in ice‐cold M‐PER mammalian protein extract reagent (Thermo Scientific) with gentleMACS tubes (Miltenyibiotec). Solubilized proteins were centrifuged at 14 000*g* in a microcentrifuge (4°C) for 10 minutes. Supernatants were separated using 10% SDS‐polyacrylamide gel electrophoresis and transferred to Immobilon P membranes (Millipore). Membranes were blocked for 1 hour at room temperature in Tris‐buffered saline (10 mmol/L Tris‐HCl, pH 7.4, 140 mmol/L NaCl) containing 0.1% Tween‐20 (TTBS) and 5% BSA (Sigma), and then incubated with 5% BSA in TTBS solution containing primary antibodies for 12 to 16 hours at 4°C. Membranes were washed in TTBS, and bound antibodies were visualized with Alkaline phosphatase‐coupled secondary antibodies and Lumi‐Phos WB (Pierce) according to the manufacturer's directions. 10 μg total protein was loaded on gels to analyze total PTEN, total and phosphoSer^473^Akt (both from Cell Signaling), and β‐actin (Sigma).

### Statistical Analysis

Data were expressed as means±SEM. Parametric tests were used after verification that variables in each group were normally distributed. One‐way analysis of variance (ANOVA) was performed to compare hypoxia‐exposed mice to normoxia‐exposed control WT mice and to compare PTEN knockout mice to WT mice during normoxia and hypoxia. *P*‐values <0.05 were considered significant for the initial ANOVA and Tukey's multiple comparison test was then used (*P*<0.05). Since only 2 time points were examined in this study and experiments were conducted separately, significant changes over time were not analyzed. For studies described in Figure 7 (7 days hypoxia followed by 3 weeks normoxia), 2‐tailed *t* test analyses were conducted. *P*‐values <0.05 were considered statistically significant.

## Results

### PTEN is Inactivated in Pulmonary Vascular SMCs in Human Pulmonary Hypertension

**Figure 1.**
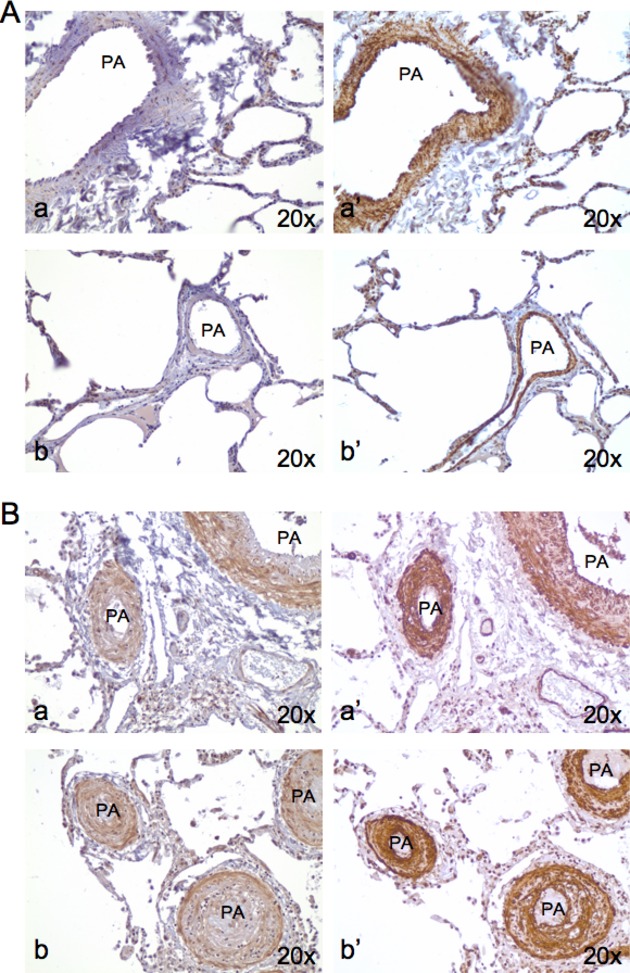
Inactivation of PTEN in SMCs of human pulmonary hypertensive patients. Serial sections of lung tissue from normal controls (A) or pulmonary hypertensive patients (B) were immunohistochemically stained for phosphorylated PTEN (a and b) or αSMA (a' and b') (brown reaction color). Representative images from 2 independent normals and pulmonary hypertensive patients are shown. Tissues from patients with PH associated with CREST syndrome (Ba) and primary PH (Bb) are shown. n=3 (normal controls); n=6 (pulmonary hypertension). SMCs indicates smooth muscle cell; αSMA, smooth muscle alpha actin; PH, pulmonary hypertension; PA, pulmonary artery.

It was previously demonstrated that PTEN is dysregulated in a variety of experimental PH models, including monocrotaline‐ and hypoxia‐induced PH as well as PH secondary to left heart failure.^35–36^ We sought to determine whether PTEN inactivation is observed in human pulmonary hypertension. Sections of human lesions were immunostained for expression of phospho‐PTEN, which is the inactivated form of the enzyme. As shown in Figure 1A, normal lungs showed low levels of phospho‐PTEN. In contrast, all samples analyzed from pulmonary hypertensive patients showed marked increases in phospho‐PTEN staining, and importantly this was largely localized to medial smooth muscle (Figure 1B; sections from patients with PH associated with CREST syndrome and primary PH only shown). These data suggest that chronic inactivation of PTEN is observed in human PH lesions. Consistent with these data and previous studies,^35^ rats exposed to chronic hypoxia demonstrated chronic PTEN inactivation selectively in SMCs, associated with increased Akt phosphorylation (Figure S1).

### Selective Inactivation of *PTEN* in SMCs Induces Pulmonary Hypertension and Hypersensitivity to Hypoxia

**Figure 2.**
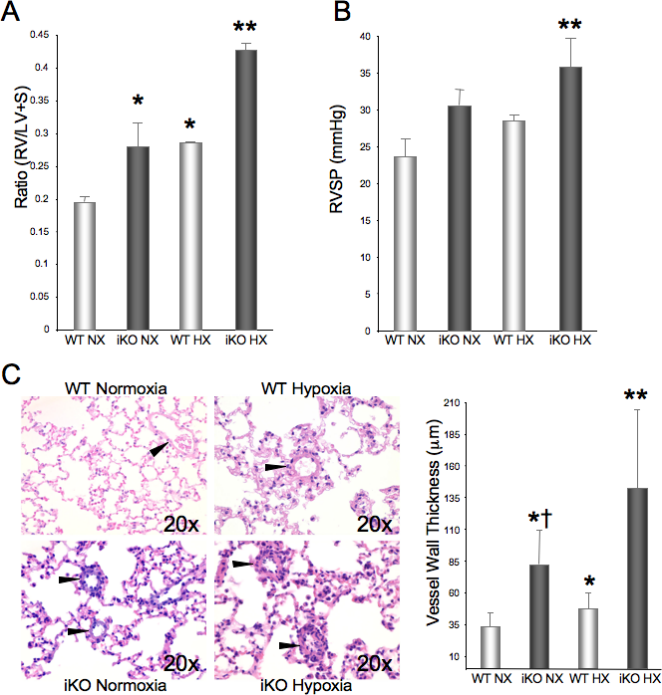
Selective inactivation of PTEN in SMC induces pulmonary hypertension and hypersensitivity to hypoxia. WT and PTEN iKO mice were treated with tamoxifen for 5 days then exposed to hypoxia (HX) for 4 weeks or maintained under normoxic conditions (NX). N=8. A, Right ventricular hypertrophy (right ventricle [RV]/left ventricle [LV]+septum [S]). B, Right ventricular systolic pressure (RVSP). C, Left—Representative H&E staining of lung sections from WT (top panels) and PTEN iKO (bottom panels) mice under normoxia (left panels) or hypoxia (right panels) showing increased wall thickness of small pulmonary arteries (arrowheads). Right—Quantification of vessel wall thickness. *Different from WT NX; **different from WT NX, iKO NX, and WT HX; †different from WT HX; *P<0.05. SMC indicates smooth muscle cell; WT, wild type; iKO, inducible knockout.

To determine if SMC‐specific PTEN loss is sufficient to induce PH, we generated mice to selectively and inducibly delete *PTEN* in SMCs (PTEN iKO; Figure S2). WT and PTEN iKO mice were treated with tamoxifen followed by exposure to hypobaric hypoxia for 4 weeks (Figure S2). Control WT and PTEN iKO mice were treated similarly, but were kept at Denver ambient pressure. Under normoxia, PTEN iKO mice exhibited elevated RVH (Figure 2A) and a nonstatistical trend for elevated RVSP (Figure 2B) compared to WT mice. These changes in PTEN iKO mice under normoxia were similar to WT mice exposed to chronic hypoxia. Unlike other animal models, exposure of WT mice to chronic hypoxia is associated with relatively minimal elevations in pulmonary arterial pressure and pulmonary vascular remodeling. In contrast, following chronic hypoxia, PTEN iKO mice displayed a much more severe PH phenotype as determined by RVH and RVSP (Figure 2A and 2B), suggesting that depleting PTEN specifically in SMC is sufficient to induce PH and PTEN inactivation synergizes with the hypertensive effects of hypoxia resulting in more severe PH.

### SMC Depletion of *PTEN* Synergizes With Hypoxia to Promote Histological Changes Associated With Advanced PH Combined With Sustained Vascular Cell Proliferation

**Figure 3.**
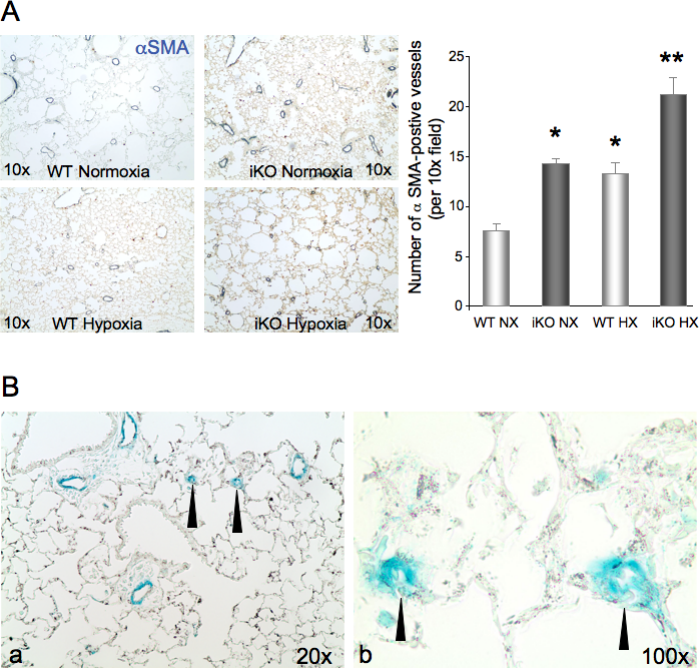
Increased distal artery muscularization in hypoxic PTEN iKO mice. A, Lung tissues from WT (left panels) and PTEN iKO (right panels) mice maintained in normoxia (top panels) or exposed to 4 weeks of hypoxia (bottom panels) were immunohistochemically stained for αSMA (gray‐blue reaction color). Left—representative staining. Right—αSMA‐positive vessel density was quantitated as described in Methods and Results. N=6; *different from WT NX; **different from WT NX, iKO NX, and WT HX; *P<0.05. B, Lung tissue from 4‐week hypoxia‐exposed PTEN iKO mice was whole mount stained for X‐Gal (blue reaction color); histological sections from these tissues were analyzed for β‐Galactosidase activity. b, is a higher magnification image of (a). iKO indicates inducible knockout; WT, wild type; αSMA, smooth muscle alpha actin; NX, normoxic; HX, hypoxia.

PH progression is associated with pulmonary arterial medial hypertrophy, adventitial thickening, muscularization of normally nonmuscular arteries, and in advanced stages intimal proliferation and obstruction/obliteration of medium and small pulmonary arteries. Similar to the observed hemodynamic changes, compared to WT mice, PTEN iKO mice maintained in normoxia exhibited increased vessel wall thickness and some muscularization of nonmuscular arteries as determined by αSMA staining; these changes were comparable to WT mice exposed to hypoxia (although vessel wall thickening was more pronounced in normoxic PTEN iKO mice) (Figures 2C and 3A). Importantly, hypoxia exposure of PTEN iKO mice resulted in significantly increased vessel wall thickness combined with extensive muscularization of nonmuscular pulmonary arterioles (Figures 2C and 3A). The cellular origin of muscle‐like cells investing previously nonmuscular arterioles has remained a controversial issue with migration of resident SMCs, recruitment and differentiation of resident fibroblasts and/or circulating monocytes, and transdifferentiation of endothelial cells proposed to account for this muscularization.^38^ One reason for this controversy is the use of αSMA to identify newly muscularized vessels. However, αSMA can be induced in other cell types. A fate‐mapping approach based on temporally regulated βGal knock‐in selectively in differentiated SMCs was used to assess the contribution of resident SMCs to distal muscularization. We found the majority of accumulated cells were βGal(+) and therefore derived from mature SMCs (Figure 3B), establishing that migration of resident differentiated SMCs is a dominant contributor to this muscularization.

**Figure 4.**
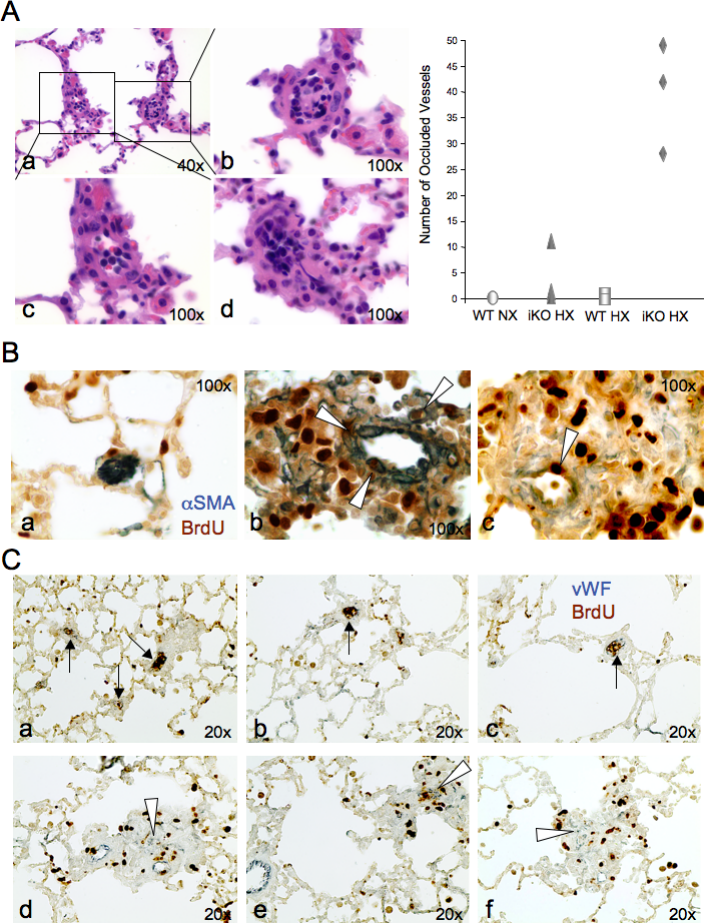
SMC‐specific depletion of PTEN collaborates with hypoxia to promote complex pulmonary vascular lesions. A, Left—Representative H&E images of occluded vessels in lung sections from PTEN iKO mice exposed to hypoxia for 4 weeks. “b and c,” are higher magnification images of “a.” Right—Total numbers of occluded vessels per condition were quantitated; each symbol represents an individual animal; n=3. B, Representative images from PTEN iKO mice exposed to hypoxia for 4 weeks showing αSMA‐positive occlusive intimal lesions of small pulmonary arteries (a) and marked vascular wall remodeling consisting of intimal, medial, and adventitial thickening and proliferating αSMA‐positive and αSMA‐negative cells (b and c). Arrowheads: BrdU‐positive replicating SMCs. C, Representative images from PTEN iKO mice exposed to hypoxia for 4 weeks showing plexiform‐like lesions. a through c, occlusive intimal lesions marked by endothelial hyperproliferation (arrows). d through f, perivascular proliferative lesions with the appearance of vWF‐positive vascular channels (arrowheads). iKO indicates inducible knockout; αSMA, smooth muscle alpha actin; BrdU, bromo‐deoxyuridine; SMCs, smooth muscle cells; vWF, von Willebrand factor; WT, wild type; NX, normoxic; HX, hypoxia.

A major disadvantage of using mouse models of PH is their inability to replicate more severe pathological findings observed in human PH patients, such as vessel occlusion. Although *PTEN* depletion alone promoted similar hemodynamic and histological changes as observed in WT mice exposed to hypoxia, we only very rarely observed occluded pre‐capillary arterioles in these conditions. The combination of PTEN iKO and hypoxia, however, consistently resulted in extensive vessel occlusion (Figure 4A). In addition, complex vascular lesions similar to lesions observed in human PH patients were observed, including αSMA‐positive occlusive intimal lesions of small pulmonary arteries and marked vascular wall remodeling consisting of intimal, medial, and adventitial thickening and proliferating αSMA‐positive and αSMA‐negative cells (Figure 4B). Finally, we observed occlusive intimal lesions marked by endothelial hyperproliferation and perivascular proliferative lesions with the appearance of vWF‐positive vascular channels similar to plexifom‐like lesions described in human PH (Figure 4C). These findings suggest that alteration of the PTEN pathway may be critical for PH progression similar to that seen in humans.

**Figure 5.**
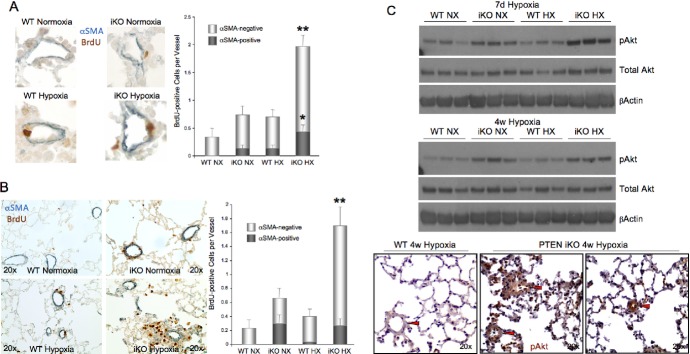
SMC‐specific depletion of PTEN collaborates with hypoxia to promote increased vascular cell proliferation. WT and PTEN iKO mice were maintained under normoxic condition or exposed to hypoxia for 7 days (A) or for 4 weeks (B). Lung sections were double immunohistochemically stained for αSMA (gray‐blue reaction color) and BrdU (brown reaction color). Left—representative stain. Right—replicating αSMA‐positive SMCs and αSMA‐negative non‐SMCs were quantitated independently as described in Methods and Results. N=6; *different from WT NX, iKO NX, and WT HX SMCs; **different from WT NX, iKO NX, and WT HX non‐SMCs;* P*<0.05. C, Top—Western analysis for phospho‐ and total Akt in whole lung tissue from WT or PTEN iKO mice maintained for 7 days or 4 weeks in normoxic (NX) or hypoxic (HX) conditions. β‐Actin was used as a loading control. Each lane represents an individual mouse. Bottom—Lung sections were immunohistochemically stained for phosphoAkt (brown reaction color). Representative stains from WT (left) and PTEN iKO mice after 4 weeks of hypoxia exposure. N=6; red arrowheads=small pulmonary arteries. SMC indicates smooth muscle cell; WT, wild type; iKO, inducible knockout; αSMA, smooth muscle alpha actin; BrdU, bromo‐deoxyuridine.

Vascular cell proliferation was analyzed by BrdU incorporation at early and late time points during the progression of PH. Compared to normoxic WT mice, following 7 days of hypoxia, no differences in proliferation of both αSMA‐positive SMCs and αSMA‐negative cells were observed in vessels of hypoxic WT mice and of normoxic PTEN iKO mice (Figure 5A). Importantly, the combination of PTEN loss and hypoxia increased proliferation of SMCs as well as αSMA‐negative cells (Figure 5A). At 4 weeks, PTEN iKO hypoxic mice showed a decline in SMC proliferation. However, the synergistic increase in non‐SMC proliferation remained high in these mice under chronic hypoxia (Figure 5B). These data suggest that sustained proliferation of vascular cells other than SMCs is critical for complex pulmonary vascular lesion formation. In addition to the synergistic effect of hypoxia and PTEN loss on cell proliferation, we detected similar effects on activation of Akt after 7 days of hypoxia (Figure 5C); phosphorylation of Akt remained high in PTEN iKO mice after 4 weeks. Phospho‐Akt localized predominantly to the pulmonary vasculature and in particular, medial SMCs (Figure 5C). In contrast, we found no differences in signal transducer and activator of transcription 3 (STAT3) activation or hypoxia‐inducible factor 1α (HIF1α) levels, both implicated in vascular remodeling in human PH and in experimental PH models^39–40^ (not shown). Thus, SMC‐specific PTEN depletion combined with hypoxia is one of very few mouse models that recapitulates the pathological characteristics of human PH.

### *PTEN* Depletion Combined With Hypoxia Induces a Synergistic Increase in Macrophage Accumulation and Sustained IL‐6 Production

**Figure 6.**
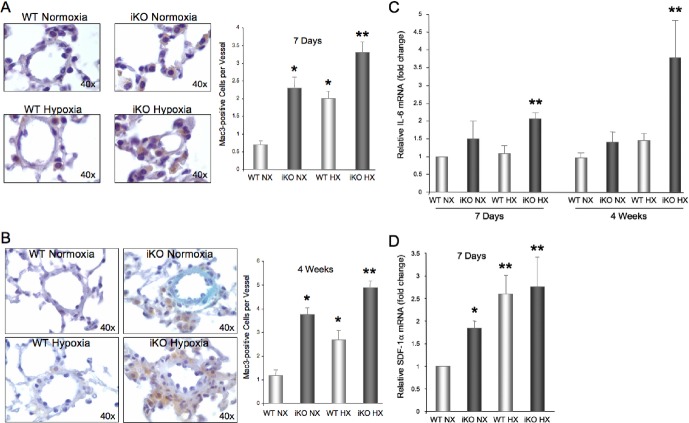
Increased macrophage accumulation in hypoxic PTEN iKO mice. WT and PTEN iKO mice were maintained under normoxia or exposed to hypoxia for 7 days (A) or for 4 weeks (B). Lung sections were immunohistochemically stained for the macrophage marker, Mac3 (brown reaction color). Left—representative stain. Right—macrophage accumulation was quantitated as described in Methods and Results. **Different from WT NX, iKO NX, and WT HX;* P*<0.05. C and D, Quantitative PCR analysis for IL‐6 (C) and SDF‐1α (D) mRNAs in lungs from WT and PTEN iKO mice maintained in normoxia or exposed to hypoxia for 7 days or 4 weeks. Actin was used for normalization of cDNA. N=6; *different from WT NX; **different from WT NX and iKO NX;* P*<0.05. iKO indicates inducible knockout; WT, wild type; NX, normoxic; HX, hypoxia; IL, interleukin; SDF‐1α, stromal cell‐derived factor‐1alpha.

While resident vascular cell proliferation contributes to the progression of PH, inflammatory cell recruitment has been shown to be a critical component of vessel remodeling and PH pathogenesis.^9^ In agreement with previous studies,^12–13^ we found that hypoxia induced an early and sustained infiltration of macrophages in WT mice (Figure 6A and 6B). This was associated with increased lung expression of the chemokine, SDF‐1α (Figure 6D), previously shown to be involved in hypoxia‐induced PH progression.^13^ A similar accumulation of macrophages was observed in normoxic PTEN iKO mice, which also exhibited increased lung SDF‐1α levels (Figure 6A and 6D). Combined hypoxia and PTEN loss resulted in an additive increase in macrophage accumulation that was sustained under chronic hypoxia (Figure 6A and 6B). While lung SDF‐1α levels remained high in all mice exposed to chronic hypoxia, levels declined over time in normoxic PTEN iKO mice (not shown) suggesting SDF‐1α is essential for the hypoxic recruitment of macrophages, but not the enhanced susceptibility for advanced lesion formation observed with the combination of PTEN loss and hypoxia. A previous report showed that lung‐specific overexpression of the cytokine, IL‐6 in combination with hypoxia promoted the development of severe PH characterized by advanced vasculopathic lesions similar to those observed in human PH patients.^41^ Macrophage infiltration has been shown to amplify tissue cytokine production, in particular IL‐6, and macrophage depletion results in decreased IL‐6 production in the setting of pulmonary hypertension.^42^ While we found no changes in IL‐6 mRNA expression in normoxic PTEN iKO mice and chronically hypoxic WT mice compared to normoxic WT mice, there was a synergistic increase in lung IL‐6 mRNA expression in response to hypoxia in PTEN iKO mice that preceded the development of PH and a further increase in chronically hypoxic PTEN iKO mice (Figure 6C). This synergistic increase correlated with increased macrophage accumulation, enhanced hemodynamic changes, and advanced lesion formation in hypoxic PTEN iKO mice. Collectively, the data suggest that hypoxia promotes macrophage accumulation prior to the development of PH, in part through induction of SDF‐1α. Macrophage infiltration combined with SMC PTEN depletion results in enhanced IL‐6 production, possibly contributing to hyperproliferation of resident vascular cells and complex vascular lesion formation.

### Transient Hypoxia Exposure Promotes Irreversible PH Progression in PTEN iKO Mice

**Figure 7.**
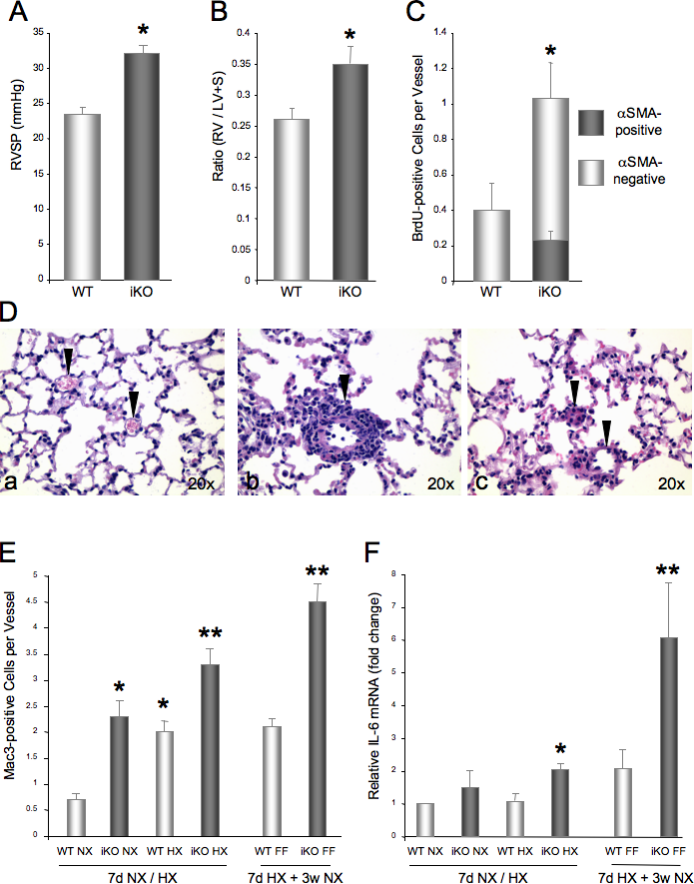
Persistence of PH progression in PTEN iKO mice after removal from hypoxia. WT and PTEN iKO mice were exposed to hypoxia for 7 days and returned to ambient pressure for 3 weeks. N=4. A, Right ventricular systolic pressure (RVSP). B, Right ventricular hypertrophy. Means±SEM. A and B,—*different from WT; *P<0.05. C, Lung sections were double immunohistochemically stained for αSMA and BrdU and replicating αSMA‐positive SMCs and αSMA‐negative non‐SMCs were quantitated independently. *Different from WT non‐SMCs;* P*<0.05. D, Representative H&E staining of lung sections from WT (a) and PTEN iKO (b and c) mice showing increased wall thickness of small pulmonary arteries (arrowheads). E, Lung sections from WT and PTEN iKO mice following 7 days hypoxia/normoxia or mice exposed to hypoxia for 7 days and returned to normoxia for 3 weeks were immunohistochemically stained for Mac3 and macrophage accumulation was quantitated. F, Quantitative PCR analysis for IL‐6 mRNA in lungs from WT and PTEN iKO mice maintained as in (E). Actin was used for normalization of cDNA. E and F,—*different from WT NX; **different from WT NX, iKO NX, and WT HX (7 days NX/HX; left 4 bars of each graph) or different from WT FF (7 days HX+3 weeks NX; right 2 bars of each graph); *P*<0.05. Seven days NX/HX and 7 days HX+3 weeks NX groups were statistically analyzed separately. PH indicates pulmonary hypertension; iKO, inducible knockout; WT, wild type; αSMA, smooth muscle alpha actin; BrdU, bromo‐deoxyuridine; SMCs, smooth muscle cells; PCR, polymerase chain reaction; IL, interleukin; NX, normoxic; HX, hypoxic; FF, feed forward; RV, right ventricle; LV, left ventricle; S, septum.

Unlike irreversible human PH, progression of PH in animal models is reversed upon removal of the hypoxic stimulus. We hypothesized that genetic pertubation of *PTEN* combined with transient exposure to hypoxia is sufficient to promote PH progression even if the hypoxic stimulus is removed. WT and PTEN iKO mice were exposed to hypoxia for 7 days followed by 3 weeks of return to ambient pressure (Figure S3). Whereas 7 days of hypoxia exposure was not sufficient to induce PH in WT mice at 4 weeks, PTEN iKO mice exhibited elevated RVSP and RV hypertrophy at the end of the 4‐week period (Figure 7A and 7B). These increases reached levels near those observed in PTEN iKO mice chronically exposed to hypoxia for 4 weeks. Physiological changes were accompanied by increased proliferation of non‐SMCs and significant vessel remodeling (Figure 7C and 7D). Macrophage accumulation in WT mice after the 4‐week period was similar to the accumulation observed following an acute 7‐day exposure to hypoxia, but did not further increase following removal of the hypoxic stimulus. In contrast, macrophages continued to accumulate in the lungs of PTEN iKO mice, even in the absence of hypoxia (Figure 7E). Similar to chronic hypoxia, transient exposure to hypoxia in the setting of *PTEN* inactivation resulted in a synergistic increase in lung IL‐6 mRNA expression (Figure 7F) that correlated with the continued accumulation of macrophages. These data suggest a feed forward model in which genetic inactivation of *PTEN* selectively in SMCs enhances susceptibility to a second stimulus (eg, hypoxia), which promotes progression of PH that is irreversible even if the secondary stimulus is removed.

## Discussion

Patients with severe irreversible PH exhibit complex pulmonary vascular arteriopathies that are associated with increased inflammatory cell accumulation.^3,8^ Although the causal factors of PH have not been defined, altered vasoconstriction and vascular remodeling are major contributors to the progression of PH.^10^ While significant effort has been invested in defining the structural alterations that occur in the walls of pulmonary arteries, the signaling pathways that drive reprogramming of vascular cells are less well defined. There is strong data identifying several growth factor pathways that are critical for the progression of PH^43–46^; thus we chose to focus on the convergent downstream PTEN/PI3K pathway. Several reports have indicated that loss or inactivation of PTEN is observed in the setting of PH.^34–35,47^ Furthermore, there are reports that germline mutations in the *PTEN* gene in humans is linked to PH. For instance, case reports showed that patients with Cowden syndrome developed PH.^48^ Our data examining lung tissues from human patients with PH showed that phosphorylated, inactivated PTEN was selectively localized to the medial layer of the vessel wall, suggesting that sustained inactivation of PTEN is characteristic of human PH. To establish the causal role of PTEN inactivation in the progression of PH, we deleted *PTEN* selectively in SMCs using an inducible system, thereby precluding confounding results that could be obtained using a constitutive system. Loss of PTEN in SMCs was sufficient to induce features of PH similar to those seen in WT mice exposed to chronic hypoxia. A major finding from this study is that the combination of PTEN loss and hypoxia resulted in more severe and sustained disease, manifesting many of the histological features observed in humans. Importantly, we report one of few models of irreversible progression of PH, highlighting the importance of disruption of PTEN signaling as an essential molecular switch underlying the pathophysiology of PH.

We initially hypothesized that loss of PTEN would lead to significant vascular remodeling through increased SMC proliferation. While our data showed a sustained increase in SMC proliferation, this was not the major contributor to the synergy between PTEN loss and hypoxia stimulation. Indeed, we found the number of proliferating SMCs did not change significantly with the introduction of hypoxia, especially at later times. In contrast, under conditions of combined PTEN loss and hypoxic exposure, in which we observed a more severe PH phenotype, the most striking findings were enhanced proliferation of non‐SMCs and increased accumulation of macrophages. These findings suggest that PTEN depletion leads to an “activated SMC” that is essential for progression of a more severe PH phenotype through paracrine effects on resident vascular and recruited inflammatory cells. We propose that activated SMCs function by promoting an inflammatory environment primed for PH progression, in part through increased macrophage numbers within the remodeled vessels. Consistent with this, Vergadi et al^49^ recently reported that early recruitment of macrophages was critical for progression of PH. Establishing an inflammatory environment around remodeled vessels may also lead to dysfunction of other cells, such as endothelial cells, which are critical for plexiform lesion formation,^50^ and fibroblasts, which contribute to marked adventitial thickening.^51^ A critical component of our model that may account for the increased severity of the disease is the sustained inactivation of PTEN. This is consistent with our observations in human PH that selective and chronic inactivation of PTEN is observed in SMCs. Thus we propose that pathways likely involving contributions from inflammatory cells act to sustain PTEN inactivation, resulting in an irreversible progression of the disease in humans.

Several studies have identified IL‐6 as a critical inflammatory cytokine in the progression of PH^11,42^ and that targeted overexpression of IL‐6 in lung epithelial cells results in occluded vessel formation,^41^ similar to results with our model as well as the human disease phenotype. We postulated that interactions between activated SMCs and macrophages were critical in creating an inflammatory environment characterized by elevated IL‐6 levels. Our data support this hypothesis showing a synergistic and sustained increase in IL‐6 with the combination of hypoxia and SMC PTEN loss. At this time, however, we cannot exclude the possibility that other resident cells within the vessel wall or other recruited cells may be playing a significant role in fostering and maintaining this inflammatory environment. Stenmark's group defined the important role of fibroblasts and recruited monocyte precursors in extensive adventitial remodeling observed in pulmonary hypertensive calves.^51–53^ Other groups have identified additional immune cells, including T and B lymphocytes and dendritic cells around advanced human PH lesions^6–7,9,54^; work is underway to define the roles of these cells in our model. Similar to previous reports, our findings suggest IL‐6 is a key player in the progression of PH. However, rather than increasing IL‐6 levels through overexpression in lung epithelial cells^41^ or decreasing expression through germline deletion,^42^ our findings more closely resemble human disease as IL‐6 was increased through synergistic effects of SMC activation and macrophage recruitment, both implicated in remodeled vessels in human PH.

Another hallmark of PH is the muscularization of normally nonmuscularized arteries, which has typically been assessed by analyzing cells expressing αSMA, a marker of SMCs.^1–2,7^ It has been traditionally thought that proliferation and migration of resident SMCs accounts for distal muscularization. However, since αSMA can be induced in other cell types, in particular activated resident fibroblasts or circulating progenitor cells and/or monocytes,^38^ the precise origin of these muscle‐like cells has yet to be definitively determined. This has largely been due to the lack of a highly efficient, reliable SMC lineage tracing system. Using our genetic fate‐mapping approach, tamoxifen administration prior to hypoxia exposure genetically and permanently induced βGal expression selectively in differentiated SMMHC‐expressing SMCs.

Since tamoxifen was administered before hypoxia and then stopped, SMCs expressing SMMHC at the time of injections and their progeny were the only cells labeled with βGal throughout the experimental time period. This allowed tracking differentiated SMCs in response to hypoxia even if these cells no longer expressed SMMHC. Further, even if other cell types contributed to distal muscularization through transdifferentiation into SMMHC‐expressing muscle‐like cells, they would not be labeled with βGal since tamoxifen was not available to activate Cre recombinase during hypoxia exposure. Using a similar approach, we recently demonstrated that the vast majority of cells comprising the neointima of injured systemic vessels were derived from mature, resident SMCs.^33^ Here, we show that the large majority of cells contributing to distal muscularization do indeed arise from mature SMCs, which to our knowledge is the first report using such a lineage tracing system to demonstrate this finding.

Unlike progressive and irreversible human PH, most experimental PH models resolve once the insult is removed.^7^ We developed a modified “progression” model by removing the hypoxic insult after only 7 days of exposure to answer the following questions: (1) Is an acute hypoxic exposure adequate for chronic accumulation of macrophages? (2) Does macrophage accumulation combined with PTEN deficiency sufficiently promote an inflammatory environment that leads to severe PH phenotype even in the absence of the chronic hypoxia? (3) Does resolution occur in this progression model? Interestingly, progression of PH continued in PTEN iKO mice exposed to an acute hypoxic stimulus. Our data suggest hypoxia is required as an early stimulus, possibly to recruit an initial population of macrophages through upregulation of SDF‐1α. However, even in the absence of chronic hypoxia, a synergistic increase in macrophage accumulation and IL‐6 production was retained suggesting that cooperation between recruited macrophages and PTEN‐deficient activated SMCs was sufficient to maintain a persistent inflammatory environment leading to an irreversible, severe PH phenotype without resolution of the disease. These findings are critical in that they confirm that severe PH likely is the result of the establishment of a chronic inflammatory environment around remodeled vessels, possibly through crosstalk between activated SMCs and recruited macrophages, but independent of a continuing hypoxic insult. In summary, our data confirm the importance of SMCs in the initiation and progression of PH through cell autonomous events (eg, SMC proliferation) combined with nonautonomous events leading to enhanced proliferation of other perivascular cells, complex lesion formation, and persistent inflammation. Further, this study implicates PTEN as a critical target for therapeutic intervention, as it is chronically inactivated in the medial layer of vessels of pulmonary hypertensive patients leading to activation of many growth factor pathways that are dysregulated in PH.
